# Effects of Methane-Rich Saline on the Capability of One-Time Exhaustive Exercise in Male SD Rats

**DOI:** 10.1371/journal.pone.0150925

**Published:** 2016-03-04

**Authors:** Lei Xin, Xuejun Sun, Shujie Lou

**Affiliations:** 1 Department of Kinesiology, Shanghai University of Sport, Shanghai, China; 2 Department of Diving Medicine, Faculty of Naval Medicine, Second Military Medical University, Shanghai, China; Universidad Pablo de Olavide, Centro Andaluz de Biología del Desarrollo-CSIC, SPAIN

## Abstract

**Purpose:**

To explore the effects of methane-rich saline (CH_4_ saline) on the capability of one-time exhaustive exercise in male SD rats.

**Methods:**

Thirty rats were equally divided into to three groups at random: control group (C), placebo group (P) and methane saline group (M). Rats in M group underwent intraperitoneal injection of CH_4_ saline, and the other two groups simultaneously underwent intraperitoneal injection of normal saline. Then, the exercise capability of rats was tested through one-time exhaustive treadmill exercise except C group. Exercise time and body weight were recorded before and after one-time exhaustive exercise. After exhaustive exercise, the blood and gastrocnemius samples were collected from all rats to detect biochemical parameters in different methods.

**Results:**

It was found that the treadmill running time was significantly longer in rats treated with CH_4_ saline. At the same time, CH_4_ saline reduced the elevation of LD and UN in blood caused by one-time exhaustive exercise. The low level of blood glucose induced by exhaustive exercise was also normalized by CH_4_ saline. Also CH_4_ saline lowered the level of CK in plasma. Furthermore, this research indicated that CH_4_ saline markedly increased the volume of T-AOC in plasma and alleviated the peak of TNF-α in both plasma and gastrocnemius. From H&E staining, CH_4_ saline effectively improved exercise-induced structural damage in gastrocnemius.

**Conclusions:**

CH_4_ saline could enhance exercise capacity in male SD rats through increase of glucose aerobic oxidation, improvement of metabolic clearance and decrease of exhaustive exercise-induced gastrocnemius injury.

## Introduction

Over-exercise is defined as excessively prolonged or intense exercise, and often associated with overload training, thirsting for super-compensation, absence of recovery, or stressful competition [1–4]. When over-exercise happens in any sport, athletes would suffer from underperformance, poor appetite, sleep disturbance, mood disorders, weight loss, progressive fatigue or other abnormal physiological responses [2,5,6], even more severe systemic dysfunctions, such as rhabdomyolysis [3,7,8], immunosuppressive state [9–12], oxidative stress [13,14], kidney function failure [4,15] and systemic inflammatory response [13,16]. These detrimental effects can result from a one-time exertion or accumulative actions [2,3]. Thus, preventing and treating these pathological changes is important in avoiding the consequences of over-exercise besides scientific diagnosis and assessment.

Methane, considered as a type of clean fuel, is the main ingredient of natural gas and flammable ice, and it is also the most abundant greenhouse gas in the atmosphere [17]. Methane can be synthesized through carbohydrate fermentation in mammalian gastrointestinal tract [18]. Thus, new research demonstrated that hypoxia-induced generation of methane in eukaryotes and mitochondria is an alternative approach to methanogenesis, which can protect the organism from inflammation and oxidation [19–22]. Based on this study, Boros et al. conducted experiments on dogs both in vivo and in vitro, and their findings suggested that exogenous methane is also able to produce anti-oxidative and anti-inflammatory effects [23]. The further study from Ye et al. proved that methane saline might be an effective approach to modulate hepatic ischemia-reperfusion injury [24].

In conclusion, the anti-oxidative and anti-inflammatory profiles of methane have been demonstrated by the previous experiments of ischemia reperfusion surgery on animals. However, the protective potential of methane has not been hypothesized in muscular oxidative stress and inflammation caused by one-time exhaustive exercise. Accordingly, we set out to investigate the effect of methane on exercise ability in an animal model of one-time exhaustive exercise. The results from our study furnish evidence that injection of methane-rich saline could prolong the time of male SD rats’ exhaustive exercise, as well as decrease exhaustive exercise-induced muscular damage.

## Materials and Methods

### Animals

The research was conducted strictly in adherence to the Guide for the Care and Use of Laboratory Animals published by the US National Institutes of Health (NIH Publication no.85-23, revised in 1996) and all experimental procedures were approved by the Ethics Committee for Science Research at Shanghai University of Sport (Approval No. 2013031). Thirty male Sprague-Dawley (SD) rats weighing 265±14 g (The Experimental Animal Center of Shanghai University of Sport, Shanghai, China) were housed in group cages (5 rats per cage) and fed with food and water through free access. Room temperature varied from 20 to 24°C under a 12-h light/dark cycle (lights on: 08:00–20:00). Before any experimental operation, it took one week for all the rats to acclimate the environment.

### Methane-rich saline preparation

The methane-rich normal saline was prepared in accordance with the production of CH_4_ saline described by Ye [24].

With the help of a pressure apparatus produced by the Diving Medicine Department of the Second Military Medical University in Shanghai, methane (Baosteels Gas Co., Ltd, Shanghai, China) dissolved in 0.9% saline (TianruiPharmaceutica Co., Ltd, Ruian, China) for 3h under a pressure (0.4 MPa) to a supersaturated concentration (0.99 mM). The CH_4_ saline was produced freshly prior to experimental procedures, sterilized by gamma radiation, and stored in a normal saline bag without dead volume under an atmospheric pressure at 4°C. The CH_4_ saline was regulated at the room temperature before injection.

### Experimental protocol

All animals were familiarized with treadmill running (DSPT202, Qianjiang Technology Company, Hangzhou, China) at 0–15 m/min, 10–20 min per day for two consecutive days [12,25].

After two days’ rest, thirty rats were equally randomized to three groups (n = 10 rats per group): a control group (C), in which the rats not perform the one-time exhaustive exercise, but were injected normal saline at 10 ml/kg 5 min before the other two groups start running [24]; a placebo group (P), in which the rats performed the one-time exhaustive exercise 5 min after injected intraperitoneally normal saline at 10 ml/kg; and a methane saline group (M), in which the rats performed one-time exhaustive exercise 5 min after injected intraperitoneally CH_4_ saline at 10 ml/kg. The exhaustive exercise model was designed with minor modification of the protocol described by Kimura F. et al, and the horizontal treadmill velocity was gradually increased from 15 m/min to 35 m/min within 5–10 min [12,26–29]. It was noted that rats were monitored specially to avoid accidental injury during exhaustive exercise and placed in individual cages after exhaustive exercise. Meanwhile, C group was placed on the motionless treadmill without any other operation. Exhaustion was defined as attonity, prostrating on the ground and no evade response when gasping [28,30] and the exhaustive exercise was uniformly carried out at 09:00. The running time and weight of rats before and after exercise were recorded accurately.

### Sample collection

10 min after exhaustive exercise, 2 ml blood sample was taken from the fossa orbital venous plexus in all rats. Thereinto, 10 μl was prepared in a micropipette (The Paul Marienfeld GmbH & Co. Kg, Lauda-Königshofen, Germany) by Na-heparinized for measurement of lactate and glucose, and the remaining was added to an anticoagulation tube with heparin, centrifuged at 3500 r/min, 4°C for 10 min to isolate plasma, and stored at -80°C until use.

All rats were anesthetized with 10% chloral hydrate (300 mg/kg, injected intraperitoneally, Sinopharm Chemical Reagent Co., Ltd, Shanghai, China), followed by an abdominal dissection on an animal operation table 3 h after exhaustive exercise. Peripheral blood (5 ml) was extracted from the inferior vena cava. Then, the blood was divided and stored in the above mentioned way. The gastrocnemii were rapidly excised and washed with pre-chilled PBS (0.01 M, PH 7.4, Thermo Fisher Scientific Co., Ltd, Massachusetts, USA). The right was dipped in 4% paraformaldehyde (Sinopharm Chemical Reagent Co., Ltd, Shanghai, China) solution to do histological analysis; the left was quickly frozen in liquid nitrogen and stored in the freezer at -80°C to detect the biological indicators. All rats were comfortably sacrificed in abdominal aorta exsanguination after all samples were collected.

### Blood lactate and glucose measurement

The 10 μl micropipette (The Paul Marienfeld GmbH & Co. Kg, Lauda-Königshofen, Germany) treated by Na-heparinized was immediately transferred into a centrifuge tube which was filled with 1.5 ml Glucose/Lactate system solution (The Paul Marienfeld GmbH & Co. Kg, Lauda-Königshofen, Germany) before measurement. The blood lactate and glucose indicators were obtained from an automatic analyzer (BiosenC_line, EKF, Germany).

### Plasma biochemical indicators measurement

A series of kits, including creatine kinase (CK), urea nitrogen (UN), myeloperoxidase (MPO), malondiadehyde (MDA) and total antioxidant capacity (T-AOC), were purchased from Nanjing Jiancheng Bioengineering Institute (Nanjing, China). The optical density (OD) of reaction mixture was measured under specific wavelength with a spectrophotometer (CANY722, Shanghai Precision Instruments Co., Ltd. Shanghai, China), and then recorded to calculate concentration of biochemical indicators in plasma.

Superoxide dismutase (SOD), a marker of anti-oxidative ability, was assayed with a microplate reader (BioTek EON, BioTek Corporation, Vermont, USA) at 450 nm wavelength to detect its activity in plasma. For the SOD kit (Nanjing Jiancheng Bioengineering Institute, Nanjing, China), the intra-assay coefficient of variation (CV) was less than 5.05% and the inter-assay CV was less than 3.32%.

### Gastrocnemius homogenate and measurement

Prepare the gastrocnemius lysate under the instructions of Bio-plexlysis kit (Bio-Rad Laboratory, Inc. Hercules, California, USA). Briefly, the gastrocnemius, washed by twice in wash buffer, was cut in small pieces of about 1–3 mm^3^, then homogenized in lysis buffer containing protease inhibitor factor and 2 mM phenylmethanesulfonyl fluoride (PMSF). The lysate was centrifuged at 4500 g for 4 min at 4°C and the supernatant was aspirated with the aid of micropipette. After homogenization, the protein concentration in supernatant was determined using a Bio-Rad Protein Assay Kit (Bio-Rad Laboratory, Inc. Hercules, California, USA) according to the manufacturer’s recommendations. Then, the supernatant was stored frozen at -80°C until treated. The MPO, MDA, T-AOC and SOD in the gastrocnemius supernatant were measured in the above mentioned way.

### Cytokine detection

To analyze cytokine levels in plasma and gastrocnemius, multiplex sandwich immunoassays were performed using a Bio-plex 200 Reader and Rat Cytokine 4-plex kits (Bio-Rad Laboratory, Inc. Hercules, California, USA). Such experiment relies on Luminex technology, which can simultaneously measures interleukins (IL-1β, IL-6, IL-10) and tumor necrosis factor (TNF-α). Briefly, the anticytokine antibody-conjugated beads were added to individual wells of a flat bottom plate (96-well), 50 μl of prediluted standards or samples was added after washing, and the plate was incubated at room temperature with shaking at 850 rpm for 1 h. Then, the plate was washed and 25 μl of prediluted multiplex detection antibody was added for 30 min incubation. After washing, 50 μl of prediluted streptavidin-conjugated phycoerythrin was loaded for 10 min, followed by an additional wash and the addition of 125 μl Bio-plex assay buffer to each well. The medium was analyzed using the Bio-plex 200 system. Data is expressed as pg/ml or pg/mgprot, and the sensitivity of the kit was less than 3 pg/ml.

It is required that the protein concentration of gastrocnemius supernatant should be adjusted to 1 μg/ml in Bio-plex sample diluent with BSA (Shanghai Bio-Light Biotech Co., Ltd, Shanghai, China) before directly loaded. The supernatant was prepared as indicated above. Plasma and gastrocnemius were analyzed independently.

### Hematoxylin and eosin staining

After washed in PBS (0.01 M, pH = 7.4), the fresh gastrocnemius was quickly cut into 1 mm^3^ pieces on a dry ice-cooled pad, then placed into bottles containing 4% paraformaldelyde, fixed for at least 4 h. Thereafter, the tissue was dehydrated, hyalinized in dimethylbenzene, embedded in paraffin, cut into 8-μm thick sections and stained in hematoxylin and eosin (H&E). Images were taken with a ×20 objective using an Olympus microscope (BX53F, Olympus Co., Ltd, Tokyo, Japan). The nonquantitative histologic assessment was carried out with an image processing software (CellSens Standard, Olympus Co., Ltd, Tokyo, Japan), referring to the standard in Nonaka’s publication [31].

### Statistical analysis

Quantitative data were presented as mean ± SD and analyzed with a statistical software package (SPSS20.0; SPSS, Chicago, USA). The analysis of exercise time was determined with independent-sample t test, the value of weight was analyzed with pared-sample t test and differences in muscle biochemical parameters between groups were performed using one-way ANOVA followed by Student-Newman-Keuls or least significant difference tests. About the blood biochemical parameters, interaction of time and group was examined by two-way ANOVA. When a significant effect appeared, its all indexes were examined by paired-samples t test. In all cases, values of *P<*0.05 were considered significant.

## Results

### Effects of CH_4_ saline on treadmill running time and body weight

**Fig 1:**
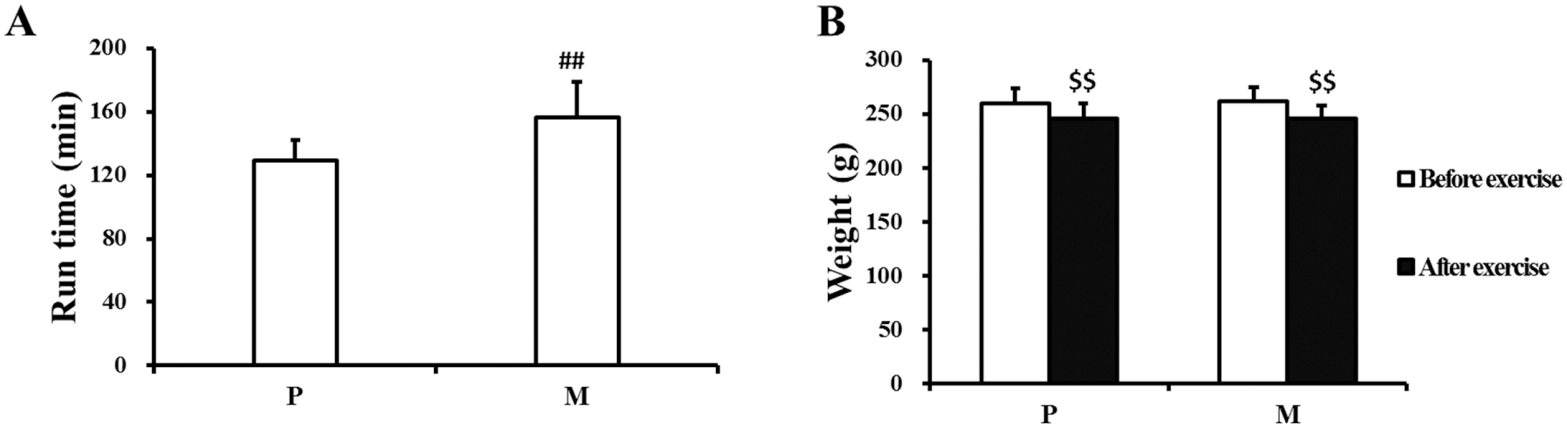
Effects of CH_4_ saline on treadmill running time and weight of male SD rats. The running time was accurately recorded in M and P groups (A). The weight of rats before and after exercise was recorded in M and P groups (B). Compared with P group, ^##^*P<*0.01 by independent-sample t test; intra-group comparison: ^$ $^*P<*0.01 by paired-sample t test. P: placebo group, M: methane saline group.

As is shown in Fig 1A, intraperitoneal injection of CH_4_ saline apparently prolonged treadmill running time of male SD rats (^##^*P<*0.01). Compared with P group, the run time was averagely increased by 27 minutes in the rats treated with CH_4_ saline. The body weight obviously decreased in both P and M groups after one-time exhaustive exercise (^$ $^*P*<0.01), but it was found that there was no significant difference between P and M groups (Fig 1B).

### Histological examination

**Fig 2:**
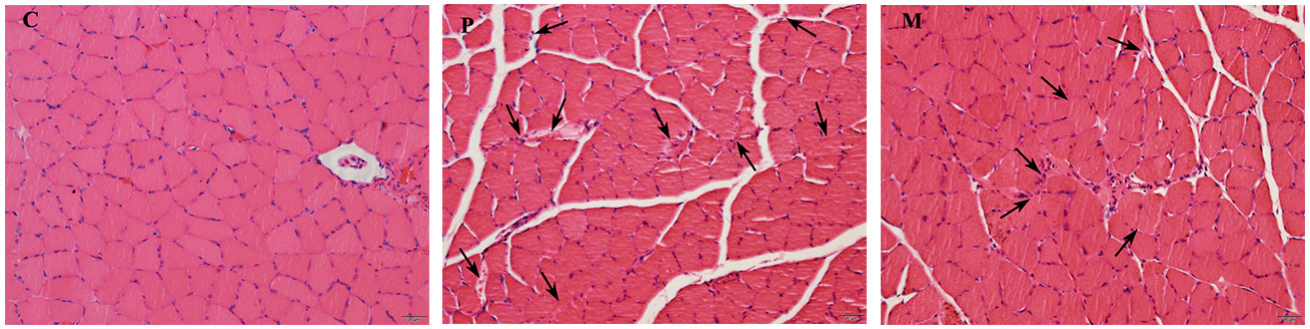
H&E stained of gastrocnemius tissue showed cell changes induced by one-time exhaustive exercise. The gastrocnemius sections were stained with hematoxylin and eosin to display the pathological changes after exercise. (C, P, M). Images were taken by Olympus microscope with a ×20 objective after H&E staining, original magnification was ×200. Arrowheads in P and M images indicated damage cells. Scale bar = 20 μm. C: control group, P: placebo group, M: methane saline group.

Fig 2 demonstrates the different degrees of gastrocnemius injuries in the three groups. The gastrocnemius sections stained with hematoxylin and eosin revealed normal histology (Fig 2C) in C group. One-time exhaustive exercise induced significant pathological changes on the cell structure of gastrocnemius in P group such as fuzzy cell borders, larger intercellular gap, and nucleus accumulation (Fig 2P); whereas, intraperitoneal injection of CH_4_ saline partly protected the gastrocnemius and weakened the gastrocnemius cell damage caused by one-time exhaustive exercise (Fig 2M).

### Effects of CH_4_ saline on the concentration of glucose and lactate in blood

**Fig 3:**
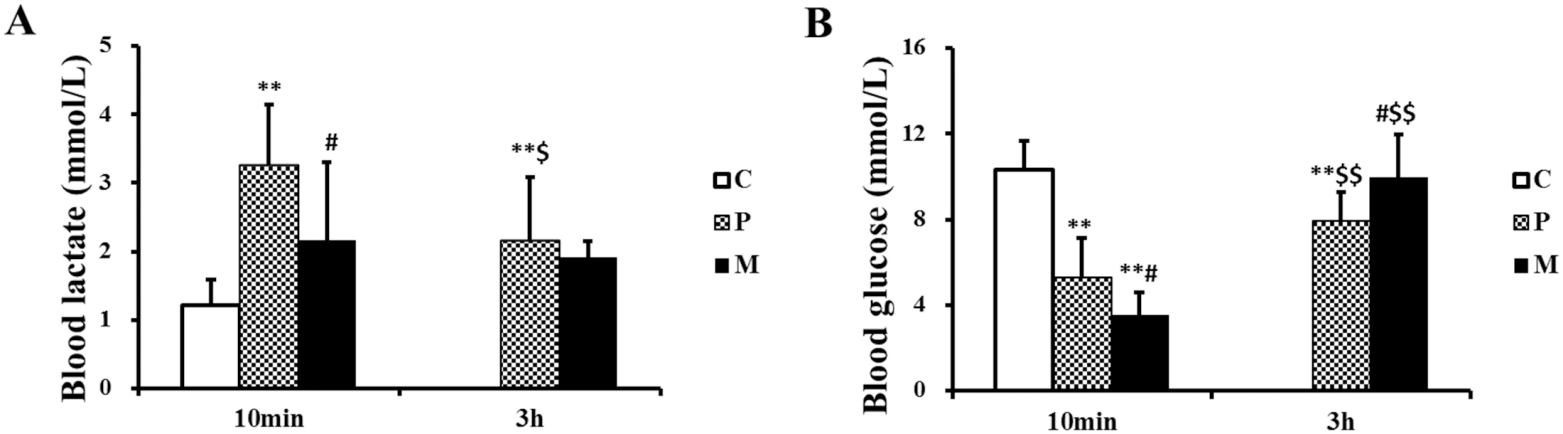
Changes of lactate and glucose in blood after one-time exhaustive exercise. The levels of blood lactate (A) and glucose (B) were measured in C, P and M groups. **P<0.01 compared with C group; ^#^*P*<0.05 compared with P group; ^$^*P*<0.05, ^$ $^*P*<0.01 compared with 10 min after exhaustive exercise. C: control group, P: placebo group, M: methane saline group.

The lactate and glucose concentration in blood was detected after one-time exhaustive exercise to study the biochemical mechanism of the exercise improvement caused by injection of CH_4_ saline, Fig 3 showed that 10 min after exhaustive exercise, there were significant elevation in blood lactate (**P<0.01) and significant reduction in blood glucose (**P<0.01) caused by exhaustive exercise when compared to C group. While, obvious changes were found between P and M groups at this time point (^#^*P*<0.05). 3 hours later, the concentration of blood lactate and glucose in P group recovered, but still had statistical difference with those in C group (**P<0.01). However, there was no significant difference in blood lactate and glucose between M and C groups 3 h after exhaustive exercise. Taken together, Fig 3 showed that CH_4_ saline injection normalized the changes of blood lactate and glucose caused by one-time exhaustive exercise.

### Effects of CH_4_ saline on the increase of CK and UN in plasma caused by exhaustive exercise

**Fig 4:**
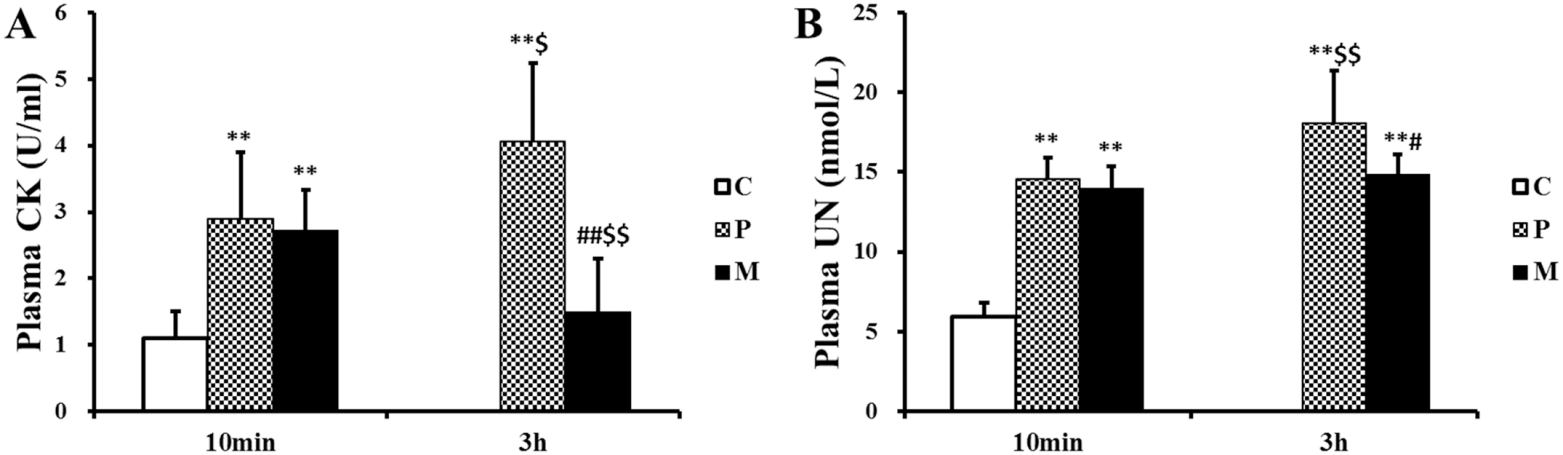
Changes of CK and UN in plasma after one-time exhaustive exercise. The levels of plasma CK (A) and UN (B) were measured in C, P and M groups. **P<0.01 compared with C group; ^#^*P*<0.05, ^##^*P*<0.01 compared with P group; ^$^*P*<0.05, ^$ $^*P*<0.01 compared with 10 min after exhaustive exercise. C: control group, P: placebo group, M: methane saline group.

Levels of CK and UN in plasma, which could respectively evaluate the extent of muscle injure and protein metabolism in rats caused by one-time exhaustive exercise, were measured by chemical colorimetry. The results in Fig 4 showed that CK and UN levels in plasma increased markedly after one-time exhaustive exercise (**P<0.01). Interestingly, CH_4_ saline injection significantly reduced the elevation of CK (^##^*P*<0.01, ^$ $^*P*<0.01) 3h after one-time exhaustive exercise (Fig 4A), and there was lower UN level (^#^*P*<0.05) in M group compared with P group 3h after one-time exhaustive exercise (Fig 4B).

### Effects of CH_4_ saline on the concentration of MDA, SOD and T-AOC in plasma and gastrocnemius

It is well-known that oxidative stress could be caused by exhaustive exercise. To study the effect of CH_4_ saline on antioxidant activity, some markers of oxidative stress in plasma and gastrocnemius, including MDA, SOD and T-AOC, were measured in this study.

**Fig 5:**
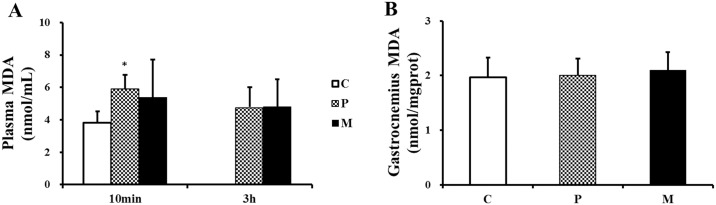
Changes of MDA in plasma and gastrocnemius after one-time exhaustive exercise. The concentration of plasma MDA was detected 10min and 3h after exercise (A), and the concentration of gastrocnemius MDA was detected 3h after exercise (B). *P<0.05 compared with C group. C: control group, P: placebo group, M: methane saline group.

10min after one-time exhaustive exercise, a significant increase in plasma MDA was seen in P group when compared with C group (*P<0.05) (Fig 5A). 3h after one-time exhaustive exercise, there was no statistical difference in plasma MDA among the three groups (Fig 5A), and the similar results were obtained in the detection of gastrocnemius (Fig 5B).

**Fig 6:**
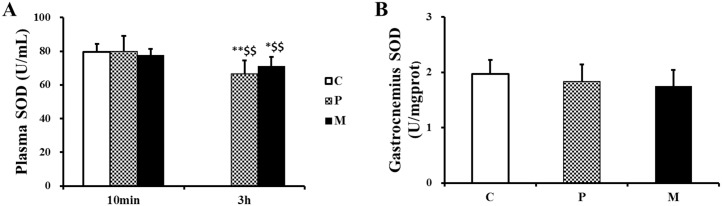
Changes of SOD in plasma and gastrocnemius after one-time exhaustive exercise. The concentration of plasma SOD was detected 10min and 3h after exercise (A), and the concentration of gastrocnemius SOD was detected 3h after exercise (B). *P<0.05, **P<0.01 compared with C group; ^$ $^*P*<0.01 compared with 10 min after exhaustive exercise. C: control group, P: placebo group, M: methane saline group.

There was no statistical difference in plasma SOD among the three groups 10 min after one-time exhaustive exercise (Fig 6A). Subsequently, the concentration of plasma SOD obviously decreased in both P (**P<0.01, ^$ $^*P*<0.01) and M (*P<0.05,^$ $^*P*<0.01) groups, but there was no significant difference between P and M groups 3h after one-time exhaustive exercise (Fig 6A), nor difference was observed in gastrocnemius at the same time (Fig 6B).

**Fig 7:**
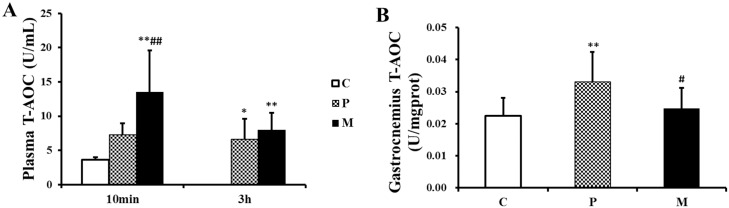
Changes of T-AOC in plasma and gastrocnemius after one-time exhaustive exercise. The concentration of plasma T-AOC was detected 10min and 3h after exercise (A), and the concentration of gastrocnemius T-AOC was detected 3h after exercise (B). *P<0.05, **P<0.01 compared with C group; ^#^*P*<0.05, ^##^*P*<0.01 compared with P group. C: control group, P: placebo group, M: methane saline group.

10 min after one-time exhaustive exercise, the T-AOC concentration in plasma in M group was significantly higher than that in P (^##^*P*<0.01) and C (**P<0.01) groups (Fig 7A). 3h after one-time exhaustive exercise, compared with C group, the concentration of T-AOC in plasma in P group significantly increased (*P<0.05), and that in M group was still higher than P group, but there was no difference between P and M groups (Fig 7A). Instead, there was a significant elevation of T-AOC in P group compared with C group (**P<0.01) when the gastrocnemii were detected. CH_4_ saline significantly reduced the elevation of T-AOC in gastrocnemius induced by one-time exhaustive exercise (^#^*P*<0.05) (Fig 7B).

### Effects of CH_4_ saline on MPO, IL-1β, IL-6, IL-10 and TNF-α levels in plasma and gastrocnemius

In order to characterize the impacts of CH_4_ saline on exhaustive exercise-induced inflammatory events, we set out to measure the levels of MPO, interleukins (IL-1β, IL-6, IL-10) and tumor necrosis factor (TNF-α) in plasma and gastrocnemius. Due to the sensitivity (≤3 pg/ml) of Rat Cytokine 4-plex Kits from Bio-Rad Laboratories, some cytokines can’t be detected including plasma IL-1β in all groups, plasma IL-6 and IL-10 in C group and TNF-α in plasma and gastrocnemius of C group.

**Fig 8:**
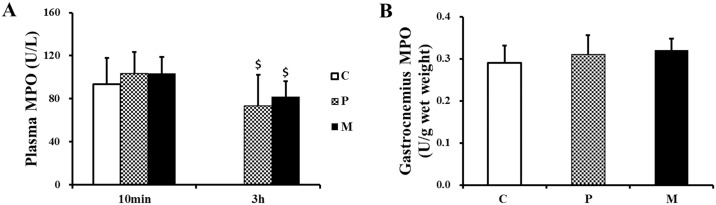
Changes of MPO in plasma and gastrocnemius after one-time exhaustive exercise. The concentration of plasma MPO was detected 10min and 3h after exercise (A), and the concentration of gastrocnemius MPO was detected 3h after exercise (B). ^$^*P*<0.05 compared with 10 min after exhaustive exercise. C: control group, P: placebo group, M: methane saline group.

As is shown in Fig 8, there was no any significant change in MPO in plasma and gastrocnemius 10 min and 3 h after one-time exhaustive exercise.

**Fig 9:**
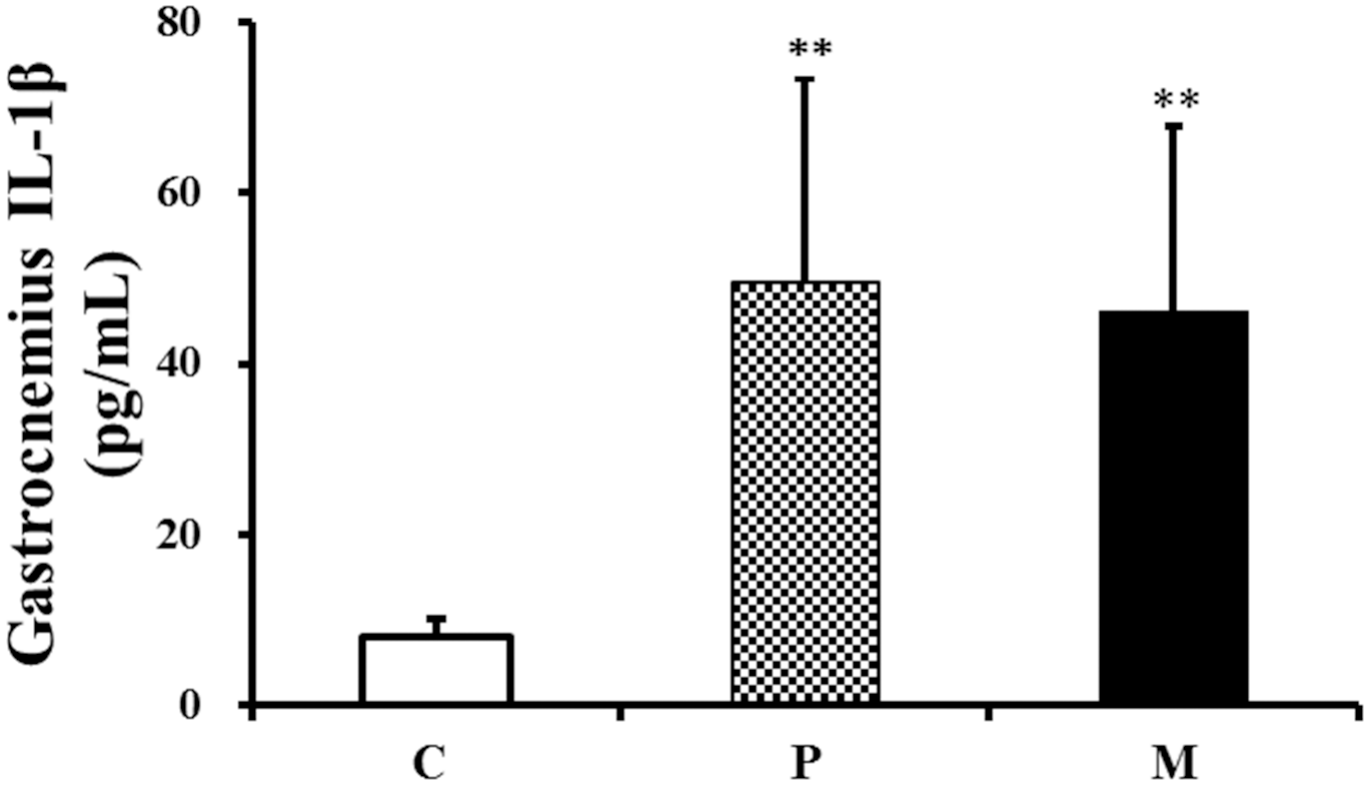
Changes of IL-1β in gastrocnemius after one-time exhaustive exercise. **P<0.01 compared with C group. C: control group, P: placebo group, M: methane saline group.

The result furnishes the evidence that the concentration of IL-1β in gastrocnemius significantly increased 3h after exhaustive exercise (**P<0.01), and injection of CH_4_ saline could not alleviate the increase of pro-inflammatory cytokine in gastrocnemius induced by exhaustive exercise (Fig 9).

**Fig 10:**
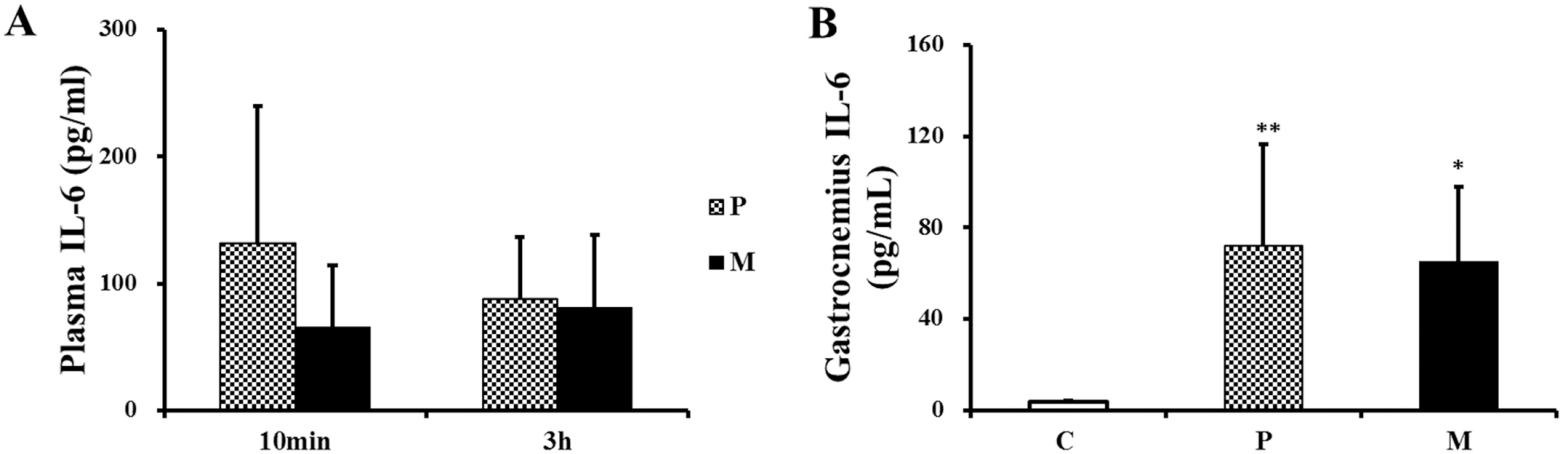
Changes of IL-6 in plasma and gastrocnemius after one-time exhaustive exercise. After exercise, the concentration of plasma IL-6 was detected at 10min and 3h (A) and the concentration of gastrocnemius IL-6 was detected at 3h (B). *P<0.05, **P<0.01 compared with C group. C: control group, P: placebo group, M: methane saline group.

As is shown in Fig 10A, there was no statistical difference in plasma IL-6 between P and M groups 10 min and 3 h after one-time exhaustive exercise. As for the detection of gastrocnemius homogenate, IL-6 concentration in C group was markedly lower than that in P (**P<0.01) and M (*P<0.05) groups 3 h after one-time exhaustive exercise (Fig 10B).

**Fig 11:**
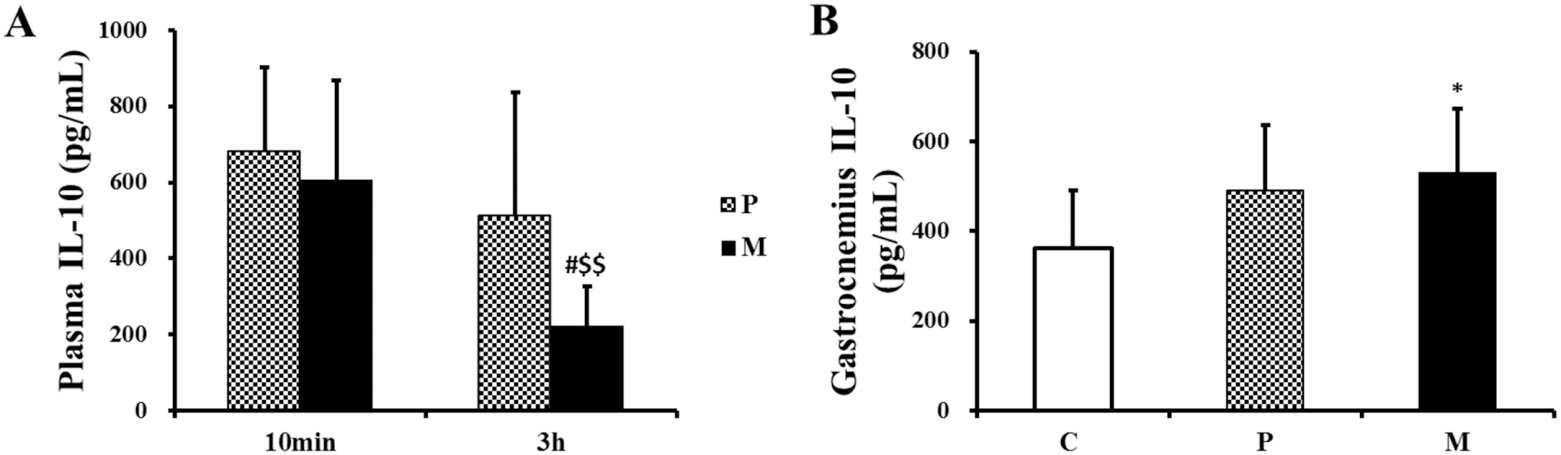
Changes of IL-10 in plasma and gastrocnemius after one-time exhaustive exercise. After exercise, the concentration of plasma IL-10 was detected at 10min and 3h (A) and the concentration of gastrocnemius IL-10 was detected at 3h (B). *P<0.05 compared with C group; ^#^*P*<0.05 compared with P group; ^$ $^*P*<0.01 compared with 10min after exhaustive exercise. C: control group, P: placebo group, M: methane saline group.

Compared with P group, the plasma IL-10 level in M group apparently decreased 3 h after one-time exhaustive exercise (^#^*P*<0.05) (Fig 11A). Interestingly, relative to P group, there was an increased tendency of gastrocnemius IL-10 in M group, but this was not a statistical difference at the same time point (Fig 11B).

**Fig 12:**
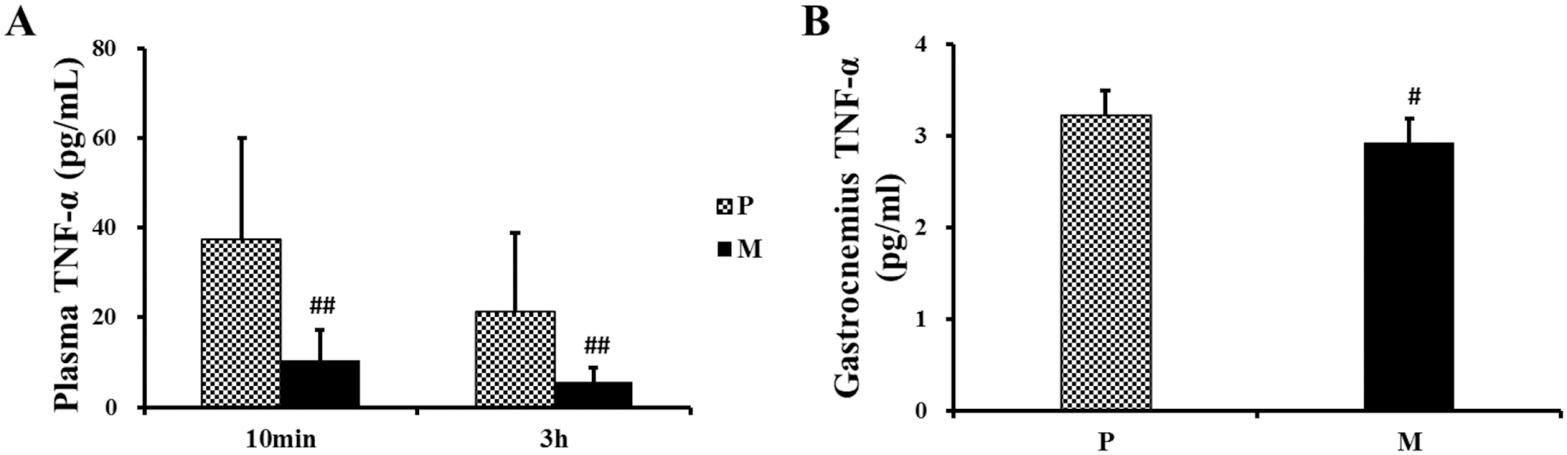
Changes of TNF-α in plasma and gastrocnemius after one-time exhaustive exercise. After exercise, the concentration of plasma TNF-α was detected at 10min and 3h (A) and the concentration of gastrocnemius TNF-α was detected at 3h (B). ^#^*P*<0.05, ^##^*P*<0.01 compared with P group. P: placebo group, M: methane saline group.

After exhaustive exercise, plasma TNF-α in M group reduced to a level lower than P group at both 10min (^##^*P*<0.01) and 3h (^##^*P*<0.01) (Fig 12A). Similarly, CH_4_ saline also reduced the level of TNF-α in gastrocnemius 3h after one-time exhaustive exercise (^#^*P*<0.05) (Fig 12B).

## Discussion

Based on Carvaho and Bedford’s studies [29,32], the exercise model we utilized is considered to be one of medium exercise intensity, which will induce rapid exhaustion after excessive exercise. In this model, the main sources of energy are from aerobic oxidation and glycolysis in this model. Toward the end of the over-exercise regime, rats demonstrated neurological fatigue and delayed responses. Meanwhile, they were not able to perform at the above mentioned speed. At the same time, biological changes occur in rats [33,34], which are parallel to all alterations in athletes after long period and medium strength exercise like marathon or triathlon.

In this study, we found that one-time exhaustive exercise could cause weight loss in male SD rats, decreases in blood glucose levels or increases in blood lactate, CK and UN concentration. In addition, there is also an elevation of inflammatory factors along with structure changes on gastrocnemius cells. Therefore, over-exercise models were successfully designed in male SD rats through one-time exhaustive treadmill running according to these biochemical measurements and previous studies. In fact, all of these markers presented in this experiment are important in evaluating athletes’ physiological functions and training effects [2,13]. On the other hand, CH_4_ saline potently modulates these physiological changes caused by over-exercise and improves exercise performance in male SD rats.

We found a significant change that treadmill running time of rats in M group was markedly prolonged, and this was related with improving skeletal blood flow and increasing percent of aerobic oxidation caused by CH_4_ saline injection. According to previous study, inhaling methane decreased the difference between intestinal intramucosal and arterial pCO_2_ levels, then improved the intestinal arterial blood flow [23]. Likewise, CH_4_ saline might increase skeletal oxygen supply and percent of energy provided by aerobic oxidation depending on the methane’s biological effect—improvement of skeletal blood flow volume. Because of these, not only the production of lactate in muscle was reduced, but also the metabolic clearance of blood lactate was advanced. On the contrary, higher level of blood lactate in P group attributed to the increase of percent of energy, which was provided by glycolysis during exercise. Meanwhile, hypoglycemia did occurred in rats of M group 10 min after a longer-time exercise, but 3 h later, the rapid recovery of blood glucose was showed up in those rats treated by CH_4_ saline. In addition, the improvement of blood flow caused by methane may increase the efficiency of hepatic gluconeogenesis in M group, which might play a role in recoveries of lactate and glucose in blood.

Urea nitrogen (UN) is an end-product of protein metabolism. On one hand, exhaustive exercise causes low glycogen which will in turn result in the elevation of ATP synthesis originated from proteolysis [35,36]; on the other hand, it has been substantiated that renal blood flow decreased during exercise, which would further lead to hypourocrinia [37]. All of these changes will increase urine nitrogen level in blood. Furthermore, protein degradation within the muscles remains elevated tendency to offer adequate fuel for the survival after exhaustive exercise, thus blood urine nitrogen level continues to increase in a short time. In our study, rats in M group finished a longer distance after CH_4_ saline injection, but the blood urine nitrogen (BUN) level of these rats is similar to that in P group; moreover, after 3 h, BUN level in M group is considerably lower than P group. CH_4_ saline might improve renal blood flow and the level of ATP generation originated from fuels other than protein, which in turn lowers the production of urea nitrogen and increases urate excretion.

Besides, creatine kinase (CK) in plasma is also a common biochemical indicator for exercise fatigue assessment and injury diagnosis [5]. A series of studies have been demonstrated that over-exercise could increase the level of plasma creatine kinase because of the changes of muscular cell permeability [38,39]; however, this elevated tendency was moderated by CH_4_ saline in the present study, which might refer to the literature of Boros et al. It is hypothesized that methane (nonpolar micromolecules) may accumulate transiently in cell membrane interfaces to influence some physicochemical properties including membrane permeability [23,40]. As is revealed by gastrocnemius H&E staining, intraperitoneal injection of CH_4_ saline significantly weakened the damage of muscular cell membrane caused by one-time exhaustive exercise. In conclusion, methane-rich saline may be available to enhance rats’ performance through maintaining the integrity of cell membrane and preventing exercise-induced exudation of creatine kinase.

Our results demonstrated that CH_4_ saline had no influence on MDA and SOD but significantly lower the level of T-AOC in gastrocnemius. Total antioxidant capacity (T-AOC), a set of endogenous antioxidants, plays an important role in free radical scavenging [41]. According to previous report, exogenous methane could effectively inhibit ROS generation of phorbol 12-myristate 13-acetate-activated polymorphonuclear (PMN) granulocytes to maintain redox homeostasis in vitro experiments [23]. On the other hand, intracellular redox homeostasis was mediated by Kelch-like ECH-associated protein 1-nuclear factor-erythroid2 p45-related factor 2-antioxidant response element (Keap1-Nrf2-ARE) signaling pathway [42], which could be activated with acute exercise in mice skeletal muscle [43]. Then, the elevated ROS due to acute exercise stress could inhibit Keap1 activity and promote Nrf2 bind to ARE region to upregulate transcription of antioxidant genes [44,45]. In our study, CH_4_ saline might attenuate exhaustive exercise- induced ROS and result in a lower basal expression of Nrf2 in gastrocnemius of M group, which further leads to a low expression of endogenous antioxidants (such as T-AOC) in gastrocnemius. However, it was inexplicable that 10 min after exhaustive exercise, CH_4_ saline enhanced plasma T-AOC in M group as compared with that in P group. Hence, further experiments are necessary to display the potential relationship between methane and Nrf2.

In addition, the anti-inflammatory effect of methane-rich saline has been observed in previous studies [24,46–48]. However, it is not clear that whether CH_4_ saline could also exert beneficial effects on protecting muscle from exhaustive exercise-induced inflammatory response. Classically, various protocols of exhaustive exercise were able to promote similar inflammatory responses, which would activate the self-protective mechanism of immune response, leading to the increase of anti-inflammatory cytokines [49]. Acute contraction-induced inflammatory damage to skeletal muscle is known to be associated with a cytokine cascade mediated by nuclear factor-kappa B (NF-kB) signaling pathway [50]. The activated pro-inflammatory transcription factor (NF-kB) could trigger the shuttling of the p65 subunit into the nucleus and then up-regulate the transcription of pro-inflammatory molecules, such as tumor necrosis factors (TNF-α) [51,52]. According to Gholamnezhad et al. reports, exercise-induced up-regulation of TNF-α mRNA in contracting skeletal muscle could stimulate type 2 lymphocytes which is in turn promotes the production of IL-10 in order to balance pro/anti-inflammatory cytokine [53–55]. Our results exactly confirmed that representative pro-inflammatory cytokines (TNF-α) were significantly reduced in rats of injected with CH_4_ saline. Meanwhile, interleukin-10 (IL-10), a downstream protein of compensatory anti-inflammatory response, was apparently reduced in plasma of M group compared with P group 3 h after exhaustive exercise. On the other hand, effects of CH_4_ saline on MPO, IL-1β, IL-6 and gastrocnemius IL-10 might be chronic but not acute. Thus, these changes haven’t been observed 10min and 3h after exercise. Even so, our results demonstrated that acute exhaustive exercise could promote inflammatory responses and then lead to an anti-inflammatory status; however, CH_4_ saline may weaken the exhaustive exercise-induced pro-inflammatory activation (TNF-α) via the NF-kB signaling system.

It is clear that there are really many open questions to be solved, especially the hemodynamic effects and metabolism of CH_4_ saline or CH_4_ in vivo. The mechanism of CH_4_ saline on the improvement of exercise capability was needed to be further explicated in wide studies. In addition, our present study primarily focused on the beneficial effects of CH_4_ saline on improving exercise ability. Inevitably, there were limitations in our study. We had insufficient time points for sample collecting after one-time exhaustive exercise, which led to the absence of continuous monitors on biochemical parameters and the lack of enough evidence to show the anti-oxidative profile of CH_4_ saline; even so, it was demonstrated that methane-rich saline could improve exercise ability in this present study.

## Conclusions

In summary, we have reported that CH_4_ saline might be an available approach to enhance the exercise ability; however, the mechanism of methane’s specific biological effects is required to be further investigated. Therefore, methane-rich saline may have broader application in the field of sport medicine.

## Supporting Information

S1 Form Form 1.tif. Animal research review committee approval form scanned copy.
(TIF)

S2 Form Form 2.tif. Animal research review committee approval form scanned copy.
(TIF)
