# Supplementary figures and images for: Effects of Methane-Rich Saline on the Capability of One-Time Exhaustive Exercise in Male SD Rats
Source: PLoS One. 2016 Mar 4;11(3):e0150925. doi: 10.1371/journal.pone.0150925 (PMC4778848; doi:10.1371/journal.pone.0150925)

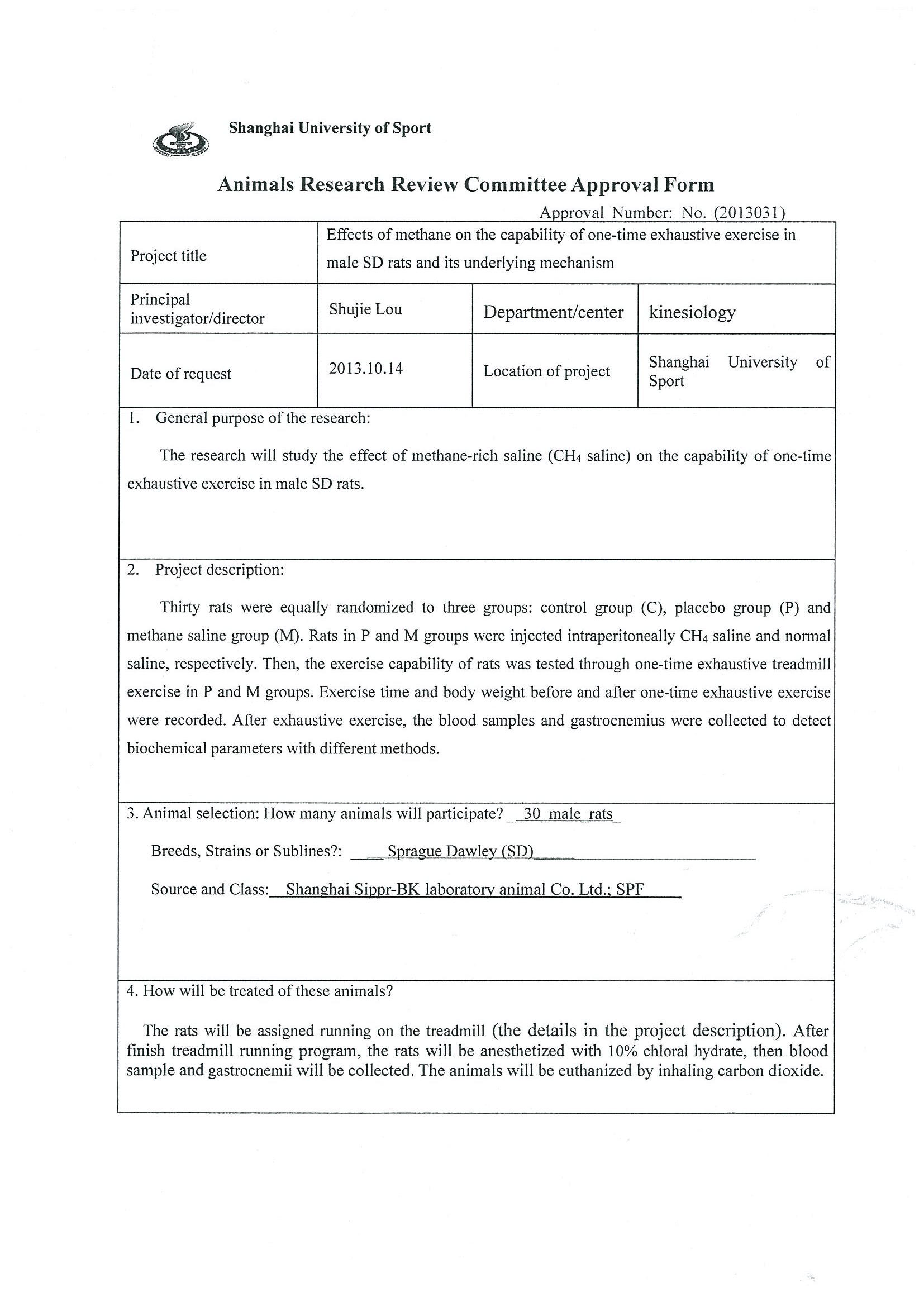

Supplement: S1 Form — Animal research review committee approval form scanned copy. (TIF) [file pone.0150925.s001.tif]

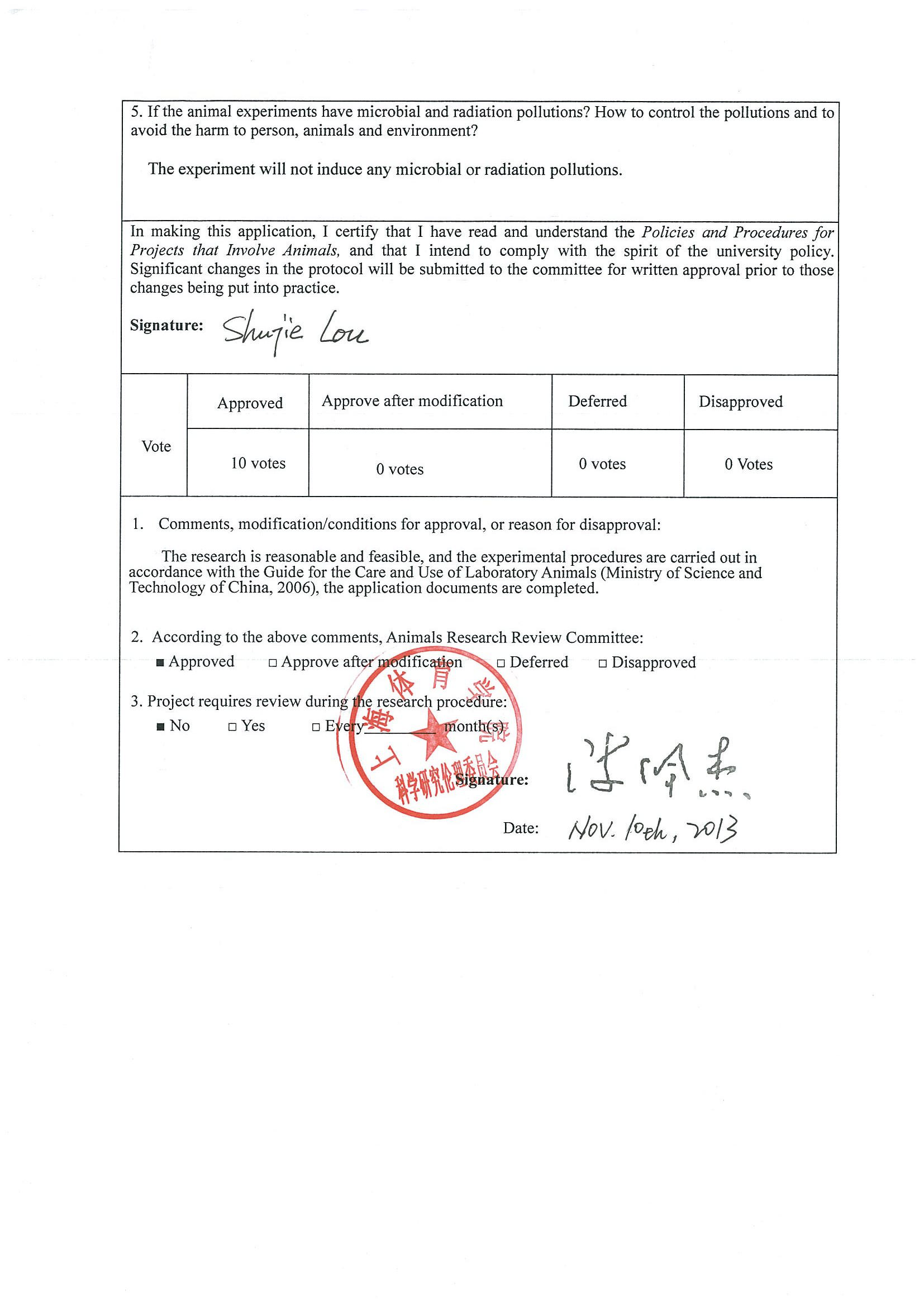

Supplement: S2 Form — Animal research review committee approval form scanned copy. (TIF) [file pone.0150925.s002.tif]
